# Development of a resource tool to assist children to adjust emotionally to the diagnosis of type 1 diabetes

**DOI:** 10.1186/1687-9856-2013-S1-P7

**Published:** 2013-10-03

**Authors:** Angie de Casanove, Surita Stipp

**Affiliations:** 1 Children’s Hospital Westmead, Australia

## 

Our previous work has shown that children recently diagnosed with type 1 diabetes (T1DM) have difficulty expressing the grief and despair that they feel in the immediate period following diagnosis. Further, many children describe feelings of isolation with their diabetes and are eager to connect with peers in a similar situation. A children’s book was therefore developed for children with newly diagnosed T1DMand their carersaimedto: 1. Create a platformfor these children to enable them to express their emotions in a safe environment with parental support. 2. To read how other children in their situation have adjusted to life with T1DM.

Over a 24 month period, approximately 60 children aged 8-12 years with T1MD attended monthly Therapeutic Support Groups at the Children’s Hospital at Westmead. The emotions and experiences during the period of T1DM were recorded as direct quotes and drawings. From this data, unique insights shed light on the complexity of the psychological process experienced by children during the time of diagnosis through exploration of specific examples it became clear that emotions such as anger, sadness and isolation were strong themes throughout the journey of diagnosis and were chosen for inclusion in the book. The collated materialprovides a greater understanding forchildren and their carersof the emotional journey experienced when first diagnosed with T1DM. The children discuss feelings and strategies regarding how to cope with injections, finger pricks, what friends can do, tips for parents and stories of hope.

The gathering of material resulted in a children’s book being published which enablesthe child to read how other children managedtheir diagnosis ofT1DM and endeavours to encourage other children to reflect. Throughout the book there are opportunities for the child to draw pictures and write how they navigated their feelings with diabetes.

In conclusion the book has been trialled by a number of Social Workers and other disciplines over the past 6 months with positive feedback from health professionals and patients. It has been used in the initial newly diagnosed sessions, counselling and groups at camps. This free book could be beneficial for rural communities who have limited psychological support as it may offer the child to feel understood while reading other children’s similar experiences.

